# Clinical experience in T cell deficient patients

**DOI:** 10.1186/1710-1492-6-9

**Published:** 2010-05-13

**Authors:** Theresa S Cole, Andrew J Cant

**Affiliations:** 1 Paediatric Immunology Dept, Ward 23, Newcastle General Hospital, Westgate Road, Newcastle, NE4 6BE, UK

## Abstract

T cell disorders have been poorly understood until recently. Lack of knowledge of underlying molecular mechanisms together with incomplete data on long term outcome have made it difficult to assess prognosis and give the most effective treatment. Rapid progress in defining molecular defects, improved supportive care and much improved results from hematopoietic stem cell transplantation (HSCT) now mean that curative treatment is possible for many patients. However, this depends on prompt recognition, accurate diagnosis and careful treatment planning.

This review will discuss recent progress in our clinical and molecular understanding of a variety of disorders including: severe combined immunodeficiency, specific T cell immunodeficiencies, signaling defects, DNA repair defects, immune-osseous dysplasias, thymic disorders and abnormalities of apoptosis.

There is still much to discover in this area and some conditions which are as yet very poorly understood. However, with increased knowledge about how these disorders can present and the particular problems each group may face it is hoped that these patients can be recognized early and managed appropriately, so providing them with the best possible outcome.

## Introduction

T cell disorders have been poorly understood until recently. Lack of knowledge of underlying molecular mechanisms together with incomplete data on long term outcome made it difficult to assess prognosis and give the most effective treatment. Rapid progress in defining molecular defects, greatly improved supportive care and much improved results from hematopoietic stem cell transplantation (HSCT) now mean that curative treatment is possible for many patients. However, this depends on prompt recognition, accurate diagnosis and careful treatment planning by experienced immunologists. Through discussion of many key T cell disorders such as: severe combined immunodeficiency, other T cell disorders, thymic disorders and disorders of lymphocyte apoptosis we hope this review will aid this process.

### Severe Combined Immunodeficiency

Severe Combined Immunodeficiency (SCID) describes a heterogeneous group of genetically determined conditions which result in lymphopenia and hypogammaglobulinemia, with inability to fight infection and early death. Four main mechanism result in SCID: defective cytokine dependent signaling in T cell pre-cursors, defective V(D)J rearrangement, defective pre-TCR or TCR signaling and premature cell death due to accumulation of purine metabolites. The most common form of SCID is the X-linked form due to mutations in genes coding for the common cytokine receptor gamma chain which is shared by the receptors for interleukin (IL)-2, IL-4, IL-7, IL-9, IL-15, and IL-21. Please see Table 1: Molecular defects that can present as SCID for details of common molecular defects and their features. Some clinical features are common to all forms of SCID whilst others are pathognomic for specific types. Recognition of the exact form of SCID is important as this influences the optimal way HSCT is performed.

**Table 1:** Molecular defects that can present as SCID.

Disease	T cells	B cells	Immunoglobulin	Other features
**Defect in cytokine signaling**				

Common γchain deficiency	↓↓	N or ↑	↓	Markedly decreased NK cells

IL7Rα deficiency	↓↓	N or ↑	↓	Normal NK cells

JAK3 deficiency	↓↓	N or ↑	↓	Markedly decreased NK cells

**Defect in VDJ recombination**				

Artemis deficiency	↓↓	↓↓	↓	

Cernunnos/XLF deficiency	↓	↓	↓	Microcephaly & radiation sensitivity

DNA ligase IV	↓	↓	↓	Microcephaly & radiation sensitivity

RAG1/2 deficiency	↓↓	↓↓	↓	

**Defects in TCR associated signaling**				

CD3δ/ε/ξ deficiency	↓↓	N	↓	Normal NK cells

CD45 deficiency	↓↓	N	↓	Normal γ/δ T cells

**Disorders of purine metabolism**				

ADA deficiency	Absent from birth or progressive ↓	Absent from birth or progressive ↓	Progressive ↓	Neurological & Radiological features

PNP deficiency	Progressive ↓	N	N or ↓	Neurological & autoimmune features

**Other defects**				

Reticular dysgenesis	↓↓	Normal or ↓	↓	Arrested myeloid maturation

↓ = decreased, ↓↓ = markedly decreased, ↑ = increased, N = normal

SCID classically presents in the first few months of life with respiratory or gastro-intestinal infections and associated failure to thrive. Often these are due to common pathogens such as Respiratory Syncytial Virus (RSV) or Parainfluenza virus causing a chronic bronchiolitic like illness or rotavirus resulting in persistent diarrhea. Opportunistic infections especially Pneumocystis jiroveci (PJP) are common. In one study one in five patients with SCID had PJP[1]. Often there is a history of cough and dyspnea for weeks or even months. A high index of suspicion is required as the organisms are rarely detected on nasopharyngeal secretions, more commonly bronchoalveolar fluid is needed. Recurrent, but treatment responsive superficial candidiasis is another important but easily overlooked diagnostic clue. Disseminated cytomegalovirus (CMV) infection is a less common but potentially devastating infection presenting with pneumonitis, hepatitis and encephalitis. Examination of a child with SCID often reveals them to be wasted with tachypnea and intercostal recession with or without crepitations or rhonchi on auscultation. Lymphoid tissue is sparse or absent, a sign best observed by looking for cervical or inguinal nodes as demonstrating the absence of tonsillar tissue is difficult in small infants. The full blood count can greatly aid diagnosis, yet is often overlooked. An absolute lymphocyte count <2.7 × 10^9^/L is abnormal in an infant and suggests SCID which can then be confirmed on lymphocyte phenotyping. In SCID there is a severe reduction in T cell numbers with variable B and NK cell numbers. Immunoglobulin levels may be unhelpful due to the presence of residual maternal IgG and the ability of some infants with SCID to make non-functional IgM. Isohemagluttinins may be useful in determining in vitro IgM production. Lymphocytes usually fail to proliferate after mitogen stimulation. Chest X-ray will show an absent thymus. Hematopoietic stem cell transplantation is the definitive treatment; trials of gene therapy are ongoing, with mixed results. Supportive treatment should be instigated as soon as the diagnosis is suspected, including Cotrimoxazole prophylaxis, antifungal prophylaxis and immunoglobulin replacement.

A significant proportion of SCID patients present with an eczematous rash, hepatosplenomegaly and lymphadenopathy, features quite different from "classical" SCID, however the other features of SCID are usually also present. In some families carrying the same molecular defect one child can present with classical SCID while another can present with this variant. In most cases one of the well recognized molecular defects is present but restricted clones of aberrant lymphocytes are present. These either arise in the patient because the defect preventing lymphocyte maturation is not complete (Omenn's syndrome) or from maternal lymphocytes that have crossed the placenta and engrafted in the child (SCID with maternal fetal engraftment). There is considerable similarity between the clinical features in these conditions, though they are usually more severe in Omenn's syndrome. The skin is red and scaly with a characteristic leathery feel, the axillary and inguinal lymph nodes are large and rubbery, often reaching three to four centimeters in diameter. Hair and, in particular eyebrows are absent. Patients with this condition are more at risk of Staphylococcus aureus and Pseudomonal infection following colonization of the abnormal skin. Useful immunopathologic clues include the presence of very high percentage of DR positive T cells without any naïve T cells, oligoclonality on TCR V beta studies or T cell receptor serotyping, eosinophilia and a raised IgE level (due to a Th4 skewing of aberrant T cells). SCID with maternal fetal engraftment is diagnosed by identifying lymphocytes of maternal origin, by XX/XY karyotyping if the patient is male or by tissue typing or the use of hypervariable DNA probes.

Abnormalities in purine metabolism, due to adenosine deaminase (ADA) deficiency or purine nucleoside phosphorylase (PNP) deficiency result in the accumulation of toxic metabolites that damage T, B and NK cells. ADA deficiency typically presents slightly earlier than other forms of SCID, lymphocyte numbers may be normal at birth but fall rapidly. Bony changes seen on chest X-ray are often pathognomic, with flaring of the anterior rib ends, blunting of the inferior angle of the scapula and pelvic dysplasia. Babies with ADA SCID are often irritable and sometimes have pneumonitis or hepatitis without an infectious cause, highlighting that this is a metabolic disease. PNP deficiency results in slower onset of immunodeficiency, initially affecting T cells but later damaging B cells. Neurological features including a characteristic dysarthria and spastic diplegia are often present by diagnosis which may not be until 4-5 years of age. Severe viral infection such as adenovirus pneumonia or gastroenteritis together with autoimmune features, including hemolytic anemia, thrombocytopenia and thyroiditis are the most common clinical features resulting from the immune defect[2].

All organisms need to have mechanisms for repairing naturally occurring DNA damage, such as occurs after defective replication, ultraviolet light or naturally occurring ionizing radiation. The immune system has adapted this system to enable T and B cell DNA to be broken, rearranged and rejoined so as to produce the huge number of receptors need to generate a comprehensive adaptive immune repertoire. It is not surprising therefore, that when genetic defects occur in DNA repair mechanisms there are neurodevelopmental and dysmorphic features as well as a defective immune response. Genetic defects that result in non-functioning proteins are embryologically lethal in DNA ligase IV mutations but not the other DNA repair defects. Hypomorphic mutations give rise to a range of protein function. This means that the clinical picture can vary considerably. Patients with DNA ligase IV and Cernunnos -XLF can present with a classical SCID picture, but also have typical phenotypic features including microcephaly, developmental delay and sun sensitivity which can provide clues to the diagnosis.

Reticular dysgenesis (RD) is a rare form of SCID due to a defect in lymphoid and myeloid differentiation. Recently a mutation in the gene coding for the mitochondrial energy metabolism enzyme adenylate kinase 2 (AK2) has been identified in six individuals with reticular dysgenesis from five independent families[3]. There is a global impairment of lymphoid maturation along with an arrest in myeloid maturation. T and B cell numbers are both low. Patients with RD present in the first few weeks of life, almost always with bacterial sepsis and neutropenia, with or without thrombocytopenia. Severe congenital neutropenia or autoimmune neutropenia are the main differential diagnoses. Bone marrow examination (which shows arrest of myeloid differentiation at the promyelocytic stage) along with low T and B cell numbers help differentiate RD from these other conditions.

HSCT is the only curative treatment for SCID. Survival has improved dramatically since it was first performed in 1968 and is now 80% for matched sibling or well matched unrelated donor transplant[4]. Improved survival is the result of many factors including: advances in understanding the mechanisms of disease, more precise matching of donor and recipient tissue types, less toxic pre-transplant chemotherapy, improved management of complications such as graft versus host disease (GvHD) and earlier diagnosis and better treatment of infections. The majority of patients have good immune function and are off all medication post transplant. A minority of patients have long term complications including: chronic graft versus host disease, recurrent, non-opportunistic infections or long term severe human papilloma virus infection[5].

### Other T cell immunodeficiencies

Partial T cell immunodeficiencies occur as a result of defects in signaling, T cell receptor gene rearrangements or thymic dysfunction. Often the defects are caused by hypomorphic gene defects allowing partial protein expression and function, albeit aberrant function so these disorders can also be associated with immune dysregulation. The variety and variability of these defects explains the wide spectrum of clinical features.

### Signaling defects

Zap-70 kinase is critical for T cell activation, transmitting signals from the CD3/TCR complex to the nucleus. Zap-70 is also crucial for intrathymic T cell development, particularly for the development of CD8 cells from double positive pre-cursors. Zap-70 kinase deficiency results in a severe reduction or absence of CD8 cells and a reduction in the proliferative response of CD4 cells, although CD4 numbers are often normal. Unlike classical SCID the thymus and lymphoid tissue may be present. It is sometimes associated with an elevated IgE level. Patients often present with diarrhea and failure to thrive with persistent viral enteritis due to enteroviruses, a diagnosis that can often be missed unless detailed viral studies are undertaken using PCR, culture and electron microscopy. A low CD8 count (less than100 cells/mm^3^) and failure of T cells to proliferate to antiCD3, reduced or absent proliferation to PHA and normal proliferation to IL-2, PMA and ionophone suggest the diagnosis which is confirmed by showing absence of Zap-70. Defects in genes coding for other signaling molecules that associate with the TCR such as the zeta chain cause a similar clinical picture or immunophenotype.

Major histocompatability complex deficiencies also cause immunodeficiency; MHC II is required to present antigen to CD4 lymphocytes, its absence results in lack of CD4 activation. It is also required in the thymus for positive CD4 lymphocyte selection. MHC II deficiency results not from defects in MHC II genes themselves but from defects in four genes that encode transcription factors required for MHC class II expression (RFXANK, RFX5, RFXAP, and CIITA)[6]. Absence of MHC II results in a variable phenotype with a later onset than SCID; affected children often present in the second year of life or later with bacterial, fungal or viral infections. Human herpes virus infections are particularly common, including human herpes virus 6 (HHV6), a pathogen often not looked for. Enterovirus encephalitis is also common, occurring in 40% of children in one review[7]. Cryptosporidial infection can result in gastro-intestinal or hepatic complications. Lymphocyte phenotyping shows CD4 lymphopenia with increased CD8 numbers resulting in a reversed CD4/CD8 ratio. Hypogammaglobulinemia is often present. MHC class II expression is absent on B cells and monocytes. HSCT is the only curative treatment but results are significantly worse than those for SCID, with European data showing 40% disease free survival for HLA matched transplants and only 20% survival for mismatched transplants[7].

MHC I is required for CD8 lymphocyte development and therefore presents with low numbers of CD8 cells. The genetic defect is usually found in genes coding for proteins that transport MHC I to the cell surface, for example TAP1 & 2, however defects in tapasin will also result in the same clinical picture. Children with MHC I deficiency present in later childhood with respiratory tract infections and bronchiectasis as well as characteristic skin ulcers with exuberant granulation tissue and rolled edges. Gastro-intestinal infection is rare. Lymphocyte subsets may be normal. Absence of MHC class I expression can be demonstrated. Treatment is targeted at respiratory disease, preventing progression of bronchiectasis. Unlike MHC class II it is not clear whether HSCT or supportive therapy is best.

CD40 ligand deficiency is the best described form of hyper IgM syndrome. Originally considered to be a B cell defect, identification of the molecular defect in 1993 revealed a T cell defect[8], later identified as a mutation in the gene encoding for CD40L glycoprotein (CD154). CD40L is expressed on activated T cells and binds to CD40 expressed on B cells and monocyte/macrophage derived cells. Lack of binding prevents immunoglobulin isotype switching by B cells as well as activation of Kupffer cells and pulmonary macrophages. The ensuing lack of IgA and IgG results in susceptibility to invasive bacterial infection. CD40L is also important for cross talk between T cells and monocyte derived cells during activation of the cell mediated immune response and therefore lack of CD40L can result in impairment of this response. Failure of pulmonary macrophage activation contributes to the risk of opportunistic infections such as PJP developing and ineffective Kupffer cells result in chronic gastro-intestinal and biliary cryptosporidial infection which results in chronic liver disease, sclerosing cholangitis, cirrhosis and hepatic malignancy from the second or third decade onwards. Many patients present in the latter months of infancy with an interstitial pneumonitis due to PJP or bacterial infections similar to those seen in patients with X-linked agammaglobulinemia. Neutropenia is also common and contributes to the propensity to recurrent infections[9]. A European survey suggested that although most patients on supportive treatment were alive at 15 years of age about half had died by their third decade[10]. Classically IgA and IgG are absent and IgM is raised, however it can be normal. The only curative treatment is HSCT although in older patients this can be complicated by reactivation of cryptosporidial disease and fulminant hepatic failure. Evaluation of the European experience found 58% survival with cure and expression of CD40L on activated lymphocytes[11]. A small number of combined liver and HSC transplants have been performed. Post HSCT deaths are related to infection, in particular cryptosporidium but also adenovirus or cytomegalovirus reactivation[11]. This poses a management dilemma as HSCT is much more successful if performed in young patients before the onset of liver disease.

Wiskott-Aldrich Syndrome (WAS), has many clinical features seen in T cell immunodeficiencies, however, the X-linked gene defect affects WASP protein which is important for actin polymerization ensuring cytoskeletal integrity as well as signaling in all cells derived from the hematopoietic stem cell. The complete WAS phenotype is associated with gene defects that result in the absence of WASP expression. It has a poor prognosis and most patients do not survive adolescence[12]. WAS is characterized by the triad of immunodeficiency, thrombocytopenia (with small platelets) and eczema, with potential for autoimmunity and malignancy. It often presents in infancy with petechia, bruising or bloody diarrhea and although low the platelet count may be greater than 50/mm^3^. Eczema tends to develop in infancy and is usually generalized rather than flexural, however, it is often so mild that WAS is not considered. Even when mild the presence of petechia within the patches of eczema is quite characteristic. The immune deficiency is progressive so infections may not be severe in the first 1-2 years of life. With time bacterial infections become pronounced, with recurrent and often discharging ear infections, pneumonias and skin sepsis. Bacterial meningitis can be seen despite vaccination as vaccine responses are short lived. Human herpes viral infections are a notable problem. Cold sores are common and more extensive than usual. Chicken pox can be devastating, with lesions progressing well beyond the usual five day period of cropping. The risk of secondary streptococcal and staphylococcal sepsis is high. Epstein Barr Virus (EBV) can produce a prolonged febrile illness with marked lymphadenopathy and hepatosplenomegaly. Cytomegalovirus and HHV6 infections are often insidious and prolonged and can be associated with vasculitis. Susceptibility to pox viruses result in severe and extensive molluscum contagiosum. Lymphocyte numbers may be normal in infancy but low T cell numbers are common by six years of age[13]. B cell numbers usually fall over time. IgG levels are usually normal, IgA can be normal or raised but IgM and isohemagglutinin levels are low. Vaccine response to protein antigens such as tetanus toxoid are usually present but there are decreased or absent responses to polysaccharide antigens such as pneumococcus. Autoimmunity can involve hemolytic anemia, vasculitis, renal disease, inflammatory bowel disease, neutropenia, dermatomyositis, recurrent angio-oedema, uveitis and cerebral vasculitis. Malignancy, usually EBV associated Non-Hodgkin's lymphoma, can develop in childhood but is more common in adolescence. Aggressive topical treatment may help the eczema. Antibacterial prophylaxis and immunoglobulin replacement may reduce the risk of bacterial infection. Aciclovir may help prevent HSV and VZV infections. Immunosuppression may be needed for the autoimmune phenomena but will increase the risk of fatal infection. Platelet transfusion are necessary for severe bleeding and splenectomy can lessen the frequency and severity of bleeding episodes, although it appears to increase the risk of fatal bacterial sepsis. Thus, for patients who do not express WASP curative treatment should be attempted. Currently HLA identical sibling or unrelated donor HSCT offers an 80% chance of cure, although care must be taken to ensure that myeloid as well as lymphoid cells are predominantly donor post HSCT as patients with a significant population of recipient myeloid cells remain at risk of thrombocytopenia and autoimmunity. Gene therapy also shows promising preliminary results but has not yet been fully evaluated.

Hypomorphic mutations in the WASP gene, allowing expression of WASP protein, result in a milder phenotype previously known as X-linked thrombocytopenia (XLT). These patients usually suffer from a bleeding tendency only in childhood and most survive in to adult life, albeit with a high chance of having an intracranial hemorrhage (ICH), 30 year ICH free survival is 36.8%[12]. Recent data suggest that whilst XLT patients suffer from less infections and eczema the long term risk of autoimmunity and malignancy may still be significant[14].

X-linked lymphoproliferative (XLP) syndrome, also known as Duncan's Disease (after the original family described) presents in a number of completely different ways. There are three common presentations. Firstly fulminant EBV infection with a hemophagocytic lymphohistiocytosis picture, resulting in fever, rash, splenomegaly, jaundice, anemia, thrombocytopenia, neutropenia, low fibrinogen, high triglycerides and hemophagocytosis on tissue specimens from bone marrow, lymph node or spleen. Secondly: dysgammaglobulinemia resembling common variable immunodeficiency, with associated sinopulmonary infections and lymphadenopathy. Thirdly: as EBV driven lymphoma which is often extranodal and involves the gut. Less commonly it can present with vasculitis, aplastic anemia or pulmonary lymphatoid granulomatosis. XLP can be divided in to two groups on a molecular basis. XLP 1 is associated with a mutation in SH2D1A, which encodes for the signaling lymphocyte activation molecule associated protein (SAP), which is essential for T cell and NK cell signaling needed to control EBV infected B cells. Absent SAP expression and a defect in the SH2D1A gene confirm the diagnosis. However, a significant proportion of XLP patients express or partially express SAP or are not found to have a defect in the SH2D1A gene despite showing the clinical phenotype. XLP 2 is associated with a gene mutation in the × linked inhibitor of apoptosis protein (XIAP). XIAP is expressed on lymphocytes, myeloid cells and NK cells and its function is to suppress apoptosis through interaction with caspases. Patients with XLP 2 have not been described as developing lymphomas but unlike XLP 1 develop splenomegaly as a prominent feature prior to EBV infection. Patients with XLP and fulminant EBV infection have a high risk of dying and should be treated aggressively if they demonstrate features of HLH with either Etoposide and steroids or Anti-thymocyte globulin, intravenous immunoglobulin (IVIG) and ciclosporin. Patients with dysgammaglobulinemia will benefit from IVIG. The only curative treatment is HSCT provided there is an unaffected HLA identical sibling donor or a well matched unrelated donor. If undertaken at a young age in a stable patient HSCT is likely to be successful in 80% of cases. Patients with features of XLP but no identifiable gene defect pose a management dilemma especially if they have not yet had EBV infection. In these cases the decision as to whether HSCT should be attempted is finely balanced.

### DNA repair defects

The DNA repair defects impact on many biological systems, not just the T and B cell V(D)J recombination process. This results in a variety of different syndromes, Cernunnos -XLF and DNA ligase IV present with a SCID like picture but many others also have a degree of T cell disorder. Ataxia Telangiectasia (AT) is one of the best known, resulting from mutations in the ATM gene which encodes the ATM protein, a protein kinase involved in meiotic recombination and cell cycle control. Patients develop a progressive cerebellar ataxia which starts soon after the onset of walking and results in most patients being wheelchair bound by 10-12 years of age. Cognitive function is usually preserved. Oculocutaneous telangiectasia normally appear between three to six years of age. Patients often have endocrinopathy with growth failure. The degree of immunodeficiency is variable, from isolated IgA deficiency to IgG deficiency and lymphopenia. Respiratory tract infections are common and repeated episodes of pneumonia can result in bronchiectasis. Prophylactic antibiotics or immunoglobulin may be useful to reduce the frequency and severity of infections. Most patients with classical AT die in early adulthood from respiratory failure or malignancy, including breast cancer, brain tumors or hematological malignancy[15]. Some patients with a milder variant of AT develop the neurological features later, appear to have less immunodeficiency than classical AT and survive further in to adulthood.

Nijmegen breakage syndrome is characterized by microcephaly, mild to moderate learning difficulties and facial features which are typically described as "bird like" - with a receding forehead and mandible with a prominent midface. Patients often also have large ears and sparse hair[16]. Growth retardation is often not obvious at birth but becomes visible by 2 years of age with shortening of the trunk more prominent than shortening of the limbs. Hypogammaglobulinemia is common with up to one third of patients having agammaglobulinemia. As with AT, sinopulmonary infection is common. CD4 lymphopenia is also frequent. The defective gene NBS1 encodes for nibrin, part of the protein complex downstream from ATM in the ATM signal transduction cascade. As with AT prophylactic antibiotics and IVIG can be useful in reducing the frequency and severity of infections. Patients are at risk of lymphoid malignancy which occurs in adolescence or early adulthood. HSCT using a modified Fanconi anemia conditioning protocol has been successful in correcting the immune defect but not the other features[17].

Immunodeficiency, centromeric instability and facial anomalies syndrome (ICF) is characterized by dysmorphic features: epicanthic folds, hypertelorism, flat nasal bridge and low set ears, together with hypo or agammaglobulinemia and branching of chromosomes 1,9 and 16 after PHA stimulation of lymphocytes which is diagnostic. A mutation in the gene coding for DNA methylation by the DNA methyltransferase DNMT3B has been described however, it has not been found in all patients with the ICF phenotype. Abnormal T cell function occurs despite normal T cell numbers with opportunistic infections such as PJP, severe viral warts and cutaneous fungal infections. It can present early in life with severe infections and failure to thrive, as a result of gastrointestinal infection or later in childhood with recurrent respiratory tract or cutaneous infections. Many will also have some degree of developmental delay in childhood. Prognosis is poor, particularly for those that present in infancy with a combination of infections, gastrointestinal problems and failure to thrive[18]. Immunoglobulin replacement can be useful in reducing the number of infective episodes for those less severely affected but HSCT should be considered for those with the severe form of the disease.

### Immune - osseous dysplasias

Cartilage Hair Hypoplasia (CHH) is an autosomal recessive metaphyseal chondrodysplasia associated with mutations in the ribonuclease mitochondrial RNA processing (RMRP) gene. CHH is one of four distinct skeletal disorders associated with RMRP gene defects, the others being metaphyseal dysplasia without hypotrichosis, kyphomelic dysplasia and anauxetic dysplasia. The degree of extraskeletal manifestations varies with each of these disorders and more recently mutations that cause severe immunodeficiency without skeletal changes have also been identified[19]. Within the diagnosis of CHH there is wide variety in phenotype even within the same family. Typical features include short limb dwarfism with flaring of the lower rib cage, a prominent sternum and bowing of the legs with very short and hyperextensible fingers due to ligamentous laxity. Hair is fine and sparse including eyelashes and eyebrows. Hypoplastic nails and hypopigmented skin are also common. The degree of immunodeficiency is variable, some patients present with a SCID like picture, whilst others have recurrent respiratory tract infections. Human herpes virus infections are a particular risk with fatal disseminated varicella or EBV driven lymphoma as notable features. The immune defect is predominantly T cell in nature with reduced numbers and impaired proliferation to mitogens however humoral abnormalities have also been described. CHH can also present with immune dysregulation and features of autoimmunity including anemia or neutropenia. HSCT has been successfully used for cases presenting with severe immunodeficiency

Schimke immune osseous dysplasia also has a variable phenotype. The defective gene has been identified as SMARCAL1 which codes for a chromatin remodelling protein. It is characterized by short stature, hyperpigmented macules, lymphopenia, cerebral ischemia and nephropathy leading to renal failure. Patients have dysmorphic features with a broad nasal bridge and bulbous nasal tip as well as small teeth and fine coarse hair. Ocular abnormalities are common, including corneal opacities, myopia and astigmatism. The hyperpigmented patches are common on the trunk but can extend on to the limbs and face. Infections are frequent and can be bacterial, viral or fungal related to the lymphopenia and also neutropenia. Other hematological abnormalities include anemia and thrombocytopenia. Progressive proteinuria causing nephrotic syndrome is steroid resistant and results in renal failure requiring renal transplant [20]. Neurological development is normal until the onset of cerebral ischemic episodes which are due to progressive arteriosclerosis. Treatment options are limited, cerebral and immunological complications are not affected by renal transplant and there is difficulty balancing immunosuppression post renal transplant to avoid death from overwhelming infection. HSCT has been performed with success[21].

Schwachman Diamond Syndrome (SDS) is a multisystem disease that consists of pancreatic exocrine insufficiency, cytopenias and short stature. It is the second most common cause of exocrine pancreatic failure after cystic fibrosis[22]. Steatorrhea occurs in the majority of patients before one year of age. Cytopenias vary, with neutropenia the most common. However, neutrophils also have impaired chemotaxis meaning that recurrent serious infections occur even without significant neutropenia. Anemia and thrombocytopenia are often mild. Hypogammaglobulinemia can occur, resulting in recurrent sinopulmonary infections. Skeletal abnormalities may not be visible until after the first year of life although children with SDS are often on the lower centiles at birth. Thoracic cage and digit abnormalities are also described. Osteomalacia and osteoporosis can occur as a result of impaired vitamin D absorption. Severe eczema is frequently seen at presentation. The SBDS gene mutation has been identified in more than 90% of patients with the SDS phenotype. Although the function of the gene is still not clear, it appears to have a role in ribosome biogenesis and RNA processing[23]. Treatment is required for pancreatic insufficiency to maximize nutrition and growth. Aggressive antibiotic treatment is required for infections. With current treatment survival is in to mid-adulthood however there is a high risk of hematological malignancy which carries a poor prognosis.

### Thymic disorders

The thymus is critical for the development of a fully functional T cell repertoire. T lymphocytes are derived from common lymphoid progenitors in the bone marrow which move to the thymus and as thymocytes undergo T-cell gene receptor rearrangement followed by positive selection in the thymic cortex. Only cells that recognize MHC expressed by thymic cortical epithelial cells are selected. Following positive selection these cells mature to single positive cells (CD4 or CD8) depending on the MHC molecule recognized. Strongly self-reactive cells undergo negative selection and apoptosis. Surviving thymocytes then leave the thymus from the medulla. Defects in thymocyte development due to an abnormal thymus can result in T cell disorders with increased infections or autoimmune features.

The chromosome 22q11.2 deletion syndrome is the most common chromosomal deletion syndrome and covers an array of different phenotypes including DiGeorge syndrome. The microdeletion at 22q11.2 results in abnormal development of branchial arch derived structures including the thymus, explaining the variety of clinical features. These include: cardiac abnormalities such as tetralogy of fallot and interrupted aortic arch; dysmorphic facies (hypertelorism, short philtrum, mandibular hypoplasia and low set ears); neonatal hypocalcaemia (which can present as neonatal seizures); feeding and speech difficulties related to submucosal or overt clefts in the palate and developmental delay[24]. The immunodeficiency is highly variable and only a minority present with a SCID like picture (less than 0.5% have complete DiGeorge syndrome with absent thymus and severe T cell immunodeficiency[24]). T cell numbers are often low but proliferation to mitogens is usually normal. Immunoglobulins can be normal or low and response to vaccines can be low or absent. In particular, responses to polysaccharide antigens such as pneumococcus are abnormal in early childhood resulting in recurrent respiratory tract infections. There is also an increased incidence of autoimmunity including cytopenias, arthritis and endocrinopathy in up to 30% of patients with 22q11.2 deletion syndrome. For those with a SCID like phenotype adoptive transfer of mature T cells through bone marrow or peripheral blood mononuclear cell transplant has been successful. Despite the lack of a functional thymus to mature lymphoid progenitors there is peripheral expansion of lymphocytes providing adequate protection against infection. However, tolerance does not develop and the risk of GvHD is high[25,26]. Thymic transplant has been recently developed for patients with complete DiGeorge anomaly. These cases had either very low circulating T cell numbers (typical complete DiGeorge) or oligoclonal T cell populations and lymphadenopathy (atypical complete DiGeorge). Thymic transplant was performed on 44 patients with a good outcome. Twenty five subjects tested one year after transplant had developed polyclonal T cell repertoires with proliferative responses to mitogens[27].

CHARGE syndrome consists of coloboma, heart defects, for example Tetralogy of Fallot, atresia choanae, retarded growth and development, genital hypoplasia and ear abnormalities/deafness. Cognitive function is variable, from normal IQ to severe learning difficulties and there also appear to be associated behavioral difficulties[28]. Mutations in chromodomain helicase DNA binding protein-7 (CHD7) have been identified in up to 75% of cases. There is a significant degree of overlap between CHARGE and 22q11 deletions in terms of clinical features. Unlike 22q11 deletions where immunodeficiency is a recognized feature it is often overlooked in CHARGE. There is however, a strong association between CHARGE and immunodeficiency. This ranges from T cell lymphopenia or hypogammaglobulinemia to a SCID presentation including Omenn's syndrome[29]. Any child presenting with features of CHARGE should have their immune system evaluated.

Autoimmune polyendocrinopathy-candidiasis-ectodermal dystrophy (APECED) syndrome is a rare autosomal recessive disorder also known as autoimmune polyglandular syndrome type 1(APS1). Originally described as a triad of chronic mucocutaneous candidiasis, hypoparathyroidism and hypoadrenalism, a much wider spectrum of associated autoimmune disease is now recognized. Chronic mucocutaneous candidiasis can be variable in its severity and age of onset. Autoimmune disorders include hypothyroidism, hypoparathyroidism, hypoadrenalism, diabetes mellitus, autoimmune hepatitis, vitiligo, alopecia and malabsorption due to GI tract autoantibodies[30]. Candidiasis frequently starts in childhood with other non-specific symptoms but classical features of APECED may not become apparent until early adulthood. It is often associated with dystrophic nails and dental enamel. Hyposplenia or asplenia can occur as a result of autoimmune destruction rendering patients susceptible to pneumococcal infection. APECED results from mutations in the Autoimmune Regulator (AIRE) gene. AIRE has a role in regulating the expression and presentation of peripheral tissue antigens by the thymic medullary epitheial cells to thymocytes and is involved in the clonal deletion of self-reactive thymocytes[31]. Decreased AIRE function allows autoreactive T cells to escape the thymus in to the periphery. More recently it has been recognized that AIRE also has a role in peripheral tolerance, eliminating autoreactive T cells that have escaped negative selection in the thymus[32]. Treatment is directed towards preventing oral candidiasis through good oral hygiene and treatment of specific endocrine disorders. Pneumococcal vaccine is also recommended although death due to septicemia appears to be uncommon[32].

IPEX syndrome (immune dysregulation, polyendocrinopathy, enteropathy, X-linked syndrome) presents in infancy or early childhood. Severely affected infants present with failure to thrive, persistent watery diarrhea due to the autoimmune enteropathy and a widespread eczematous rash which is difficult to treat. Hyperglycemia can start from as early as the first week of life and may require insulin to maintain glycemic control. Thyroiditis also starts early in life and can result in hyper or hypothyroidism. Hematological abnormalities are common, including: autoimmune hemolytic anemia, thrombocytopenia and neutropenia. Other manifestations include: lymphadenopathy; hepatosplenomegaly; cholestatic hepatitis; nephropathy; seizures; vasculitis and arthritis. Some cases have multiple, severe infections including sepsis, meningitis, pneumonia and osteomyelitis[33], these appear to be related to impaired barrier functions of the skin and gut rather than a specific immunodeficiency. Investigations demonstrate eosinophilia with elevated IgE but normal IgG, A and M. Autoantibodies can be found directed against a variety of different organs and cell types. Mutations have been identified in the FOXP3 gene, located in the centromeric region of the × chromosome. Absence of FOXP3 function results in the absence of regulatory T cells leading to autoaggressive lymphoproliferation. Despite being activated cells have defective IL-2, IFN-γ and TNF-α production. Treatment with immunosuppression can improve symptoms but does not achieve long term remission. The only curative treatment is HSCT. A number of cases have been described with an IPEX phenotype but no identifiable FOXP3 mutation. These have been termed IPEX-like and require similar treatment.

### Disorders of apoptosis

The immune system functions by maintaining a small population of lymphocytes specific for wide range of antigens. Pathogen recognition results in rapid proliferation of these lymphocytes. However, this mechanism must be controlled to prevent unwanted effects and malignant transformation in chronically activated cells.

Autoimmune Lymphoproliferative syndromes (ALPS) are a group of disorders characterized by abnormal apoptosis, resulting in lymphoproliferation and autoimmunity. A number of gene mutations have been identified, including fas, fas-ligand, caspase 8 and caspase 10. The spectrum of presentation is variable and like many other conditions variation occurs within a family with the same defect. The diagnosis of ALPS should be considered in any patient presenting with persistent lymphadenopathy and hepatosplenomegaly with or without evidence of autoimmunity, for example, hemolytic anemia, thrombocytopenia or neutropenia. The most common presentation is with lymphadenopathy and marked splenomegaly at around two years of age. Hepatomegaly is present in approximately 75%[34]. Occasionally patients present with only lymphadenopathy or splenomegaly. Autoimmune features are variable in nature and timing of onset. Autoantibodies are often present without evidence of clinical disease. Investigations show lymphocytosis with an increased number of TCR alfa/beta+ CD4-CD8- cells. Abnormal apoptosis can be demonstrated. Lymphadenopathy will respond to steroids but recurs on weaning. Autoimmune disease may require large amounts of immune suppression. The combination of high dose steroids, IVIG and Rituximab have been used with some success although there are concerns about prolonged hypogammaglobulinemia following the use of Rituximab. The antimalarial drug pyrimethamine-sulphadoxine has also been shown to be beneficial in reducing lymphadenopathy and cytopenias. Splenectomy has been used for resistant thrombocytopenia and anemia however post-splenectomy pneumococcal sepsis is a particular problem in ALPS[34]. Death occurs as a result of hematological malignancy, severe autoimmune disease or post-splenectomy sepsis. HSCT has been used successfully for cases that have been difficult to control using immunosuppression.

## Conclusion

Our understanding of the wide variety of T cell disorders has greatly improved in recent years. The spectrum of T cell defects is broad, from severe combined immunodeficiency to signaling defects to specific defects in lymphocyte apoptosis. This means there is massive variety in the way patients present. It is important to be aware of these conditions so the diagnosis can be considered. The patient can then be referred to an expert immunologist and investigated appropriately. It is helpful to identify the molecular diagnosis as this can contribute to understanding of prognosis and therefore the most appropriate management. It is important to recognize the infective complications that are associated with these disorders and look carefully for them. It is also necessary to understand other systems/organs that can be affected and assess the extent of damage to these, for example, lungs and liver, as this can also have an impact on potential treatment. HSCT has been successful in many of the T cell disorders and should be considered early in the disease process now that survival has improved and complications reduced. Gene therapy may become more widespread in the future as our understanding increases and results improve. Further work is still needed however in the large number of kindreds with T cell defects that have yet to be identified in order to understand their genetic defect, their risk of particular infections and therefore the best treatment for them.

## Competing interests

The authors declare that they have no competing interests.

## Authors' contributions

TSC and AJC contributed equally to the preparation of this manuscript.

All authors have read and approved the final manuscript.
